# New perpective for an old problem: extracellular vesicle based management of respiratory distress syndrome

**DOI:** 10.1080/10717544.2021.1995079

**Published:** 2021-11-03

**Authors:** Defne Engur, Abdullah Kumral

**Affiliations:** a Izmir Biomedicine and Genome Center, Izmir, 35330, Turkey; b Izmir International Biomedicine and Genome Institute, Dokuz Eylül University, Izmir, Turkey; c Division of Neonatology, Department of Pediatrics, University of Health Sciences Training Hospital, Izmir, Turkey; d Division of Neonatology, Department of Pediatrics, Faculty of Medicine, Dokuz Eylul University, Izmir, Turkey

## What are exosomes?

Exosomes are membrane-derived extracellular vesicles with an average diameter of 30–200 nanometers that are generated by budding of plasma and endosome membranes. They harbor several components including nucleic acids, proteins, lipids, and metabolites, and exert diverse effects when taken up by distant cells. This intercellular vesicle traffic can affect function and the behavior of the recipient cell (Pegtel & Gould, 2019). Exosomes provide intercellular communication in many aspects of physiologic processes including tissue homeostasis, growth and development as well as generation of immune responses. They are also involved in the pathogenesis of several diseases such as inflammatory or degenerative conditions (Kalluri & LeBleu, 2020). Molecular constituents of exosomes can reflect their cell of origin and display heterogenity in altered cellular or tissue states, thus exosomes are emerging as promising markers for diagnostic purposes (Lin et al., 2015). Manipulation of exosomal content is also possible which holds a significant potential to deliver therapeutic agents in multiple disease situations (Barile & Vassalli, 2017). Nanoparticle drug delivery technology that has developed in the last decade, became an attractive solution to improve the efficacy of therapeutic agents. However, despite the great number of proposed nanobased therapeutics, few of them are approved for clinical use. Two main barriers preventing the wide spread clinical application of nanoparticle drug delivery are cytotoxicity of the materials and rapid clearance by the immune cells (De Jong & Borm, 2008). Thus, exosomes have attained considerable attention following their discovery with their biocompatible carrier characteristics (Luan et al., 2017).

## Exosomes as drug delivery vehicles

Recent reports imply that exosomes can efficiently deliver cargo molecules for treatment of wide spectrum of diseases from cancer to neurologic problems including pulmonary conditions (Bunggulawa et al., 2018). Exosome-based drug delivery systems can be designed through exchange of cellular components creating a highly effective cell mediated drug carrier and targeting the therapeutic agent to a specific tissue, thus, desired biological activity in the recipient cell can be directed via diverse therapeutic payloads (Batrakova & Kim, 2015; Kalluri & LeBleu, 2020). Exosomes highly penetrate into tissues owing to their nano size and deformable structure (Luan et al., 2017). They enter circulation, persist a long period of time and can also evade the immune response (Luan et al., 2017). With their high stability in the bloodstream, together with their lipid bilayer and the hydrophilic core, exosomes are suitable to carry both hydrophobic and hydrophylic drugs to long distance targets (Bunggulawa et al., 2018). Exosomes can also be used to deliver post-transcriptional regulators such as microRNAs (miRNA) or interfering RNAs (siRNA) (Barile & Vassalli, 2017; Turchinovich et al., 2019).

## Use of exosomes for neonatal lung diseases

Following the experimental data on anti-inflammatory and a pro-angiogenic effects of mesenchymal stem cells (MSC) on hyperoxic lung injury and bronchopulmonary dysplasia (BPD) models, clinical trials have been commenced in human infants. In 2014, the safety and feasibility of allogeneic MSC transplantation in preterm infants at high risk for BPD have been assessed in a phase I dose-escalation trial (Chang et al., 2014). Researchers did not see any adverse effects in 2-year follow-up of these infants (Ahn et al., 2017). Standing on these results, research group has designed a phase II trial, comparing the effect of human umbilical cord derived-MSCs versus normal saline placebo (ClinicalTrials.gov identifier NCT03392467). Recently, it has been recognized that exosomes derived from MSCs also confer a therapeutic efficacy similar to MSCs themselves (Collins, 2020). MSC-derived exosomes have been shown to ameliorate the changes in the transcriptomic profile in hyperoxic lung injury model with downregulating a group of genes induced by hyperoxia and inducing a group of genes that were suppressed by hyperoxia in the rodent model of BPD (Willis et al., 2018). Although multipl preclinical studies point out the potency of MSC-derived exosomes, it still remains as a pre-clinical technology in management of neonatal lung conditions (Lesage & Thébaud, 2018).

## Surfactant for respiratory distress syndrome and current challenges

Exogenous surfactant treatment is one of the game changing advancements in neonatology that creates a major improvement in morbidity and mortality of preterm infants with respiratory distress syndrome (RDS). Administration of exogenous surfactant has been recognized as the standard therapeutic procedure for more than three decades. Both synthetic and animal-derived exogenous surfactant preparations are shown to be succesful in the prevention and treatment of RDS. Although it is well established strategy in the clinical practice, numerous studies focusing on the dose, formulation and mode of administration continiue over a dozen of years. Ongoing studies mainly address the increasing clinical demand for improvement in pulmonary distribution with better pool size, along with new techniques of delivery (Niemarkt et al., 2017).

Current practice of administration requires instillation of exogenous surfactant into patient’s lungs through a thin catheter with or without tracheal intubation. Owing to the invasive nature of this procedure, -especially considering the repeated doses that are usually needed in very preterm infants-, tremendous efforts have been devoted to identify alternative ways for surfactant delivery in the last decades, one of which is nebulisation/aerosolization. Several studies underscoring the safety of nebulized surfactant have been published, however, drawbacks regarding efficacy, especially variability of the dose delivered to peripheral airways and possible alteration of surfactant compounds remain. Intrapartum pharyngeal and intramniotic ways also have similar disadvantages (Biban et al., 2012).

Surfactant inactivation and inflammation are another main obstacles that should be considered in management of RDS. Surfactant synthesis from lungs are developmentally regulated and most often single dose of exogenous surfactant is not sufficient to maintain the pool size in small preterms and surfactant inactivation is an challenging issue especially in the presence of inflammation and in infants with limited endogenous surfactant production (Herting et al., 2000).

## Exosomes can be used for management of RDS

Beyond reducing surface tension and decreasing the work of breathing, prematurely born infants require a more comprehensive strategy for respiratory handling. Exosome based drug delivery technology could be a striking approach through providing the best practice in RDS management. First of all, exosomes could overcome challenges regarding distribution by its natural targeting ability. Exosomes could be excellent nanocarriers for exogenous surfactant delivery, and also, additional agents could be uploaded to these biological carriers such as antiinflammatory agents or molecules that inhibit surfactant degradation. Thus, enhancement of bioavailability or stability of the exogenous surfactant preparations could be retained. Since pulmonary inflammation is the major co-player in the pathogenesis of RDS, inactivation of surfactant and consecutive development of BPD, exosomes could be ideal candidates for delivery of local anti-inflammatory agents while refraining systemic adverse events of corticosteroids (Chen et al., 2019).

Another therapeutic strategy may be to equip exosomes with surfactant-associated proteins (SFTPs). Commercial surfactants contain hydrophobic surfactant proteins B and C, that essential in the formation, secretion and in the alveolar surfactant dynamics. But, hydrophilic surfactant proteins A and D are deficient in the exogenous products that are currently in clinical use (Engür & Kumral, 2012; Salgado et al., 2014). These proteins also have essential role in alveolar surfactant dynamics, besides their roles as components of the innate immune defence.

Nevertheless, a smarter solution for infants with RDS, will be through not only providing exogenous surfactant but also, induction of genes responsible for pulmonary maturation and surfactant synthesis. Synthesis and secretion of surfactant occurs in alveolar type-II epithelial cells and the genes responsible for SFTP synthesis are developmentally controlled by certain regulators. The two major factors that coordinate expression of SFTPs are, homeobox protein Nkx-2.1 (NKX2-1) and hepatocyte nuclear factor (HNF)-3. Targeted induction of the genes responsible for surfactant synthesis can be possible by transcription factors and cofactors that bind to promotor region of these genes (Jacot & Bousquet, 2003). Exosomes may be ideal carriers for targeted delivery of these genetic signals. Proper maturation of the immature lung tissue could be possible with appropriate genetic signals provided by exosomal cargo molecules. Thuswise, beyond early problems like surfactant regarding distribution, inactivation and redosing, moderate and late term complications such as maturational arrest or dysplastic development of the premature lungs could be managed.

## Conclusion

Since its discovery at 1980s, the standard regimen of surfactant treatment requires the direct instillation of the formulation into the trachea. However, studies for alternative delivery continues. Exosome-based drug delivery systems are highly effective and biocompatible drug carriers that can target the cargo molecule to the desired specific localization. Beyond surfactant and additional co-therapeutics, exosomes can deliver regulatory factors to induce genes related with lung development in preterm infants which can help maintenance of surfactant secretion and prevent bronchopulmonary dysplasia that involves maturational arrest of lungs.

## Prior presentation

No data from this manuscript were presented in a scientific meeting before.
